# Diagnostic Value and Prognostic Evaluation of Autophagy-Related Protein Expression Level in Sepsis Complicated with Acute Respiratory Distress Syndrome

**DOI:** 10.1155/2022/8920926

**Published:** 2022-03-25

**Authors:** Jia-Li Xu, Xue-Lu Yu, Xiao-Qin Yao, Li-Hong Xu, Mei Jing

**Affiliations:** ^1^Department of Emergency, Naval Medical Center, Naval Medical University, China; ^2^Department of Disease Control and Prevention, Naval Medical Center, Naval Medical University, China; ^3^Department of Pediatrics, The Second People's Hospital of Tongxiang City, China; ^4^Department of Quality Management, The 903 Hospital of Joint Logistics Support Force of the Chinese People's Liberation Army, China

## Abstract

**Objective:**

To explore the diagnostic value and prognostic evaluation of the autophagy-related protein expression level among patients with sepsis comorbid with acute respiratory distress syndrome.

**Methods:**

A total of 182 sepsis patients were admitted to Naval Medical Center from March 2016 to April 2020 and divided into the acute respiratory distress syndrome and non-ARDS groups. Immunoblotting was employed to identify the expression of autophagy-associated protein from participants' peripheral blood mononuclear cells. Multivariate linear regression analysis was used to examine the association between mortality and the protein expression in sepsis complicated with acute respiratory distress syndrome.

**Results:**

Among the 182 patients with sepsis included in this study, 82 patients had acute respiratory distress syndrome and 100 patients did not have acute respiratory distress syndrome. We observed that microtubule-related protein 1A/1B LC3II, Beclin-1, RAB7, and LAMP2 protein expression was significantly decreased in septic patients with ARDS, and p62 was significantly increased. Further receiver operating characteristic curve analysis showed that autophagy-related proteins had a high recognition ability in sepsis complicated with acute respiratory distress syndrome. LAMP2 protein was the best among them, and its specificity was up to 91.46%. In this study, 38 of the 82 patients with sepsis complicated with acute respiratory distress syndrome died, with a mortality rate of 46.34%. We found that the autophagy level was further inhibited in the patients with death, LC3II, Beclin-1, and RAB7. However, the lysosomal-associated membrane protein 2 levels in the survival patients were remarkably higher than that in the dead patients. In addition, the p62 level was lower in survival patients as well. Our results indicated age and SOFA score were the independent risk factors for mortality in septic patients with acute respiratory distress syndrome.

**Conclusion:**

The autophagy level is significantly inhibited in septic patients with acute respiratory distress syndrome, and autophagy-associated proteins LC3II, Beclin-1, RAB7, LAMP2, and p62 have good value for the diagnosis and prognosis evaluation of sepsis comorbid with acute respiratory distress syndrome.

## 1. Introduction

Sepsis refers to immune dysfunction and systemic inflammation caused by microbial infection and is a syndrome characterized by organ dysfunction and abnormal immune response to infection [1, 2]. There are more than 19 million cases worldwide every year, and the in-hospital mortality rate of sepsis patients is about 15%-25% [3, 4]. Organ dysfunction is caused by the local or systemic release of harmful mediators from the site of infection or away from the organ stimulating infection [5]. The lung is one of the most affected organs during sepsis, which may lead to acute respiratory distress syndrome (ARDS) [6]. ARDS is a clinical syndrome in critical patients, including acute respiratory failure (ARF), hypoxemia, and noncardiogenic lung oedema [7, 8]. So far, there are no effective treatments for ARDS. Therefore, it is very important to assess the biomarkers associated with sepsis-induced ARDS and to understand the molecular mechanisms of ARDS caused by sepsis.

Autophagy participates in the etiopathogenesis of ARDS. The autophagic process is a remarkably conserved cell protection activity which involves the transport of damaged proteins to lysosomes for degradation and circulation, so as to maintain the stability of cell structure, function, and metabolism. It is considered to be one of the main pathways of protein and organelle degradation and circulation [9, 10]. Recently, researchers have discovered that autophagic activation participates in the pathophysiological process of sepsis, which alleviates the excessive release of cytokines in sepsis and lung injury. This function may protect mice from the effects of acute lung injury (ALI)/ARDS that was induced by cecal ligation perforation (CLP) [11, 12], improves arterial oxygenation and vascular function, and inhibits IL-1*β* production. Autophagy levels could be identified via assessing autophagy flux levels and detecting lipid-modified forms of microtubule-related protein 1A/1B light chain 3 (LC3) and autophagosomes [13]. Elevated autophagy levels can promote the accumulation of autophagy-associated proteins LC3II and Beclin-1 [14]. Beclin-1 is an important autophagic protein, including two different subcomplexes with regulators Atg 14 and UV radiation resistance-associated gene protein (UVRAG), which responds to autophagosome formation and maturation, respectively [15]. Lysosomal-associated membrane protein 2 (LAMP2) and Ras-related protein Rab-7a (RAB7) are necessary for late autophagosomal maturation and fusion between autophagosome and lysosome [16, 17]. p62, as an autophagic biomarker, is the first protein discovered to have such an adaptor role in the autophagic process and is decomposed via autophagic stimulation [18]. Evidence has shown that there is a potential association between the suppression of autophagic activity and elevated p62 levels [19]. At present, few studies have reported the clinical significance of autophagy-associated protein expression in ARDS.

In this study, we assess the association between autophagy-related protein expression level and the diagnostic value and prognostic evaluation in sepsis patients comorbid with ARDS. Our study will provide more data for clinical understanding of the biomarkers that might be potentially used for ARDS prediction among sepsis patients.

## 2. Methods

### 2.1. Research Object

A total of 182 sepsis patients admitted to an academic medical center, Naval Medical Center, from March 2016 to April 2020 were selected. Inclusion criteria include all patients that met the diagnostic criteria for sepsis in the 2012 Save Sepsis Campaign: International Guidelines for the Management of Sepsis and Septic Shock [20]. Patients in the ARDS group also met the diagnostic criteria for ARDS in the Berlin Definition. The entire sufferers were above 18 years old with complete medical records. Exclusion criteria include those infected with human immunodeficiency virus, who died within 24 hours after admission to ICU, combined with hematopoietic diseases or malignant tumors, pregnant and lactating women, combined with mental disorders, and with severe malnutrition. The inclusion and exclusion criteria were based on previously published studies [21, 22]. According to the occurrence of ARDS, the sufferers were assigned into 2 groups, including 82 patients with ARDS and 100 patients without ARDS. The present research was accepted by the Ethical Board of our hospital, and the whole sufferers offered informed consent.

### 2.2. Peripheral Blood Collection and Monocyte Separation

Within 24 hours after admission, 5 ml of fasting venous blood was harvested from the entire sufferers in the morning by vacuo venous blood collection. The density gradient centrifugation method was used to separate mononuclear cells, and 2 ml of lymph cell separation liquor was supplemented into the round-bottom test tube, and venous blood and Hanks fluid were pressed 1 : 1. The anticoagulant diluted blood was slowly added into the separation solution tube along the tube wall with a capillary straw and centrifuged in a horizontal centrifuge at room temperature (20°C) for 20 min at 2000 r/min. A capillary straw was inserted into the white cell layer at the interface, and the cells were sucked out and added 1 ml Hanks liquid, and the cells were suspended. All our blood sample collection was progressed by well-trained nurses, and the methods were based on previous similar published studies.

### 2.3. Autophagy-Related Protein Expression Detection

Western blot (WB) was employed to identify the expression of autophagy-associated proteins LC3II, Beclin-1, RAB7, LAMP2, and p62 in mononuclear cells. Lysis was performed in a cellular lysate buffering solution (1x RIPA, CST, America) for 0.5 h, followed by centrifugation at 12,000 g at 4°C for 15 min to harvest the supernate. The protein level was identified via the Bradford Assay (Bio-RAD, America); 30 *μ*g protein was treated with SDS-PAGE and afterwards moved onto PVDF film (Millipore, America). Posterior to the sealing under RT for 120 min via 5% skim milk involving 0.1% Tween 20, it was cultivated nightlong via the first antisubstance at 4°C. Anti-mouse primary antibodies of LC3II, Beclin-1, RAB7, LAMP2, and p62 (1 : 1000) were obtained from Abcam (Cambridge, UK). After incubation with an anti-mouse secondary antibody bound to horseradish peroxidase (1 : 5000, Santa Cruz Biotechnology, America), the protein was developed using an enhanced chemiluminescence reagent (Thermo Pierce, USA). Quantitative analyses were completed via the Bio-RAD Gel Doc 2000 System (America). *β*-Actin was utilized as inner control.

### 2.4. Main Outcome Measures

The main outcome of interest was ARDS incidence in sepsis patients. In addition, sepsis patients with comorbid ARDS may have increased risks of all-cause mortality.

### 2.5. Covariates

Demographic and clinical characteristics were determined at the baseline including age, gender, smoking history, body mass index (BMI), and other comorbid conditions. The other comorbid conditions included severe pneumonia, severe pancreatitis, abdominal infection, biliary infection, surgical trauma, chronic obstructive pulmonary disease (COPD), cardiac disease, chronic kidney disease, and liver cirrhosis.

### 2.6. Statistical Analysis

The data are expressed as the average ± SD, and the *χ*^2^ test is used for comparison of measurement data. Continuous variates were contrasted via two-tailed Student's *t*-test or one-way ANOVA. The contrast of nonnormal distribution data is studied via the nonparametric test of two separate specimens. A two-tailed *P* value < 0.05 had significance on statistics. Multivariate linear regression analysis was used to examine the association between mortality and the protein expression in sepsis complicated with acute respiratory distress syndrome. We used the AUC as an overall measure of discrimination of each variable. The entire statistic analysis was finished via SPSS 17.0 (America). GraphPad Prism 5 (America) was used to draw relevant pictures.

## 3. Results

### 3.1. Clinical Information Statistics

Table 1 displays characteristics for both the ARDS and non-ARDS groups. Among the 182 patients with sepsis included in this study, 82 patients had ARDS and 100 patients did not have ARDS. The means (SD) of age were 55.96 (8.49) and 58.01 (9.45) years in the ARDS and non-ARDS groups, respectively. Compared with the non-ARDS group, the patients in the ARDS group were more likely to have smoking history and higher creatinine, CRP, and APACHE II scores (*P* < 0.05). There was no remarkable diversity in comparison to other variables (*P* > 0.05) (Table 1).

### 3.2. Expression of Autophagy-Related Protein in Septic Patients with ARDS

**Figure 1:**
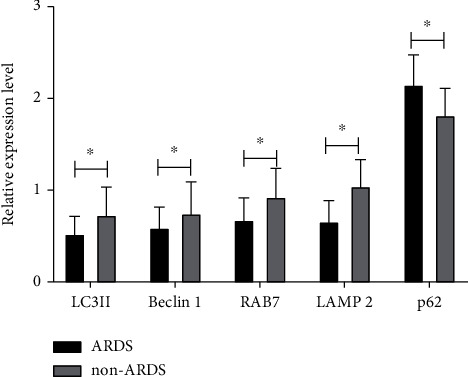
The expression of autophagy-related proteins in sepsis patients with ARDS. ^∗^*P* < 0.05.

Western blot outcomes revealed that in the peripheral blood monocytes of sepsis sufferers with ARDS, the relative expression levels of LC3II, Beclin-1, RAB7, and LAMP2 were remarkably lower in contrast to the non-ARDS group (*P* < 0.05). On the contrary, the relative expression level of p62 in the peripheral blood monocytes of sepsis sufferers with ARDS was remarkably greater in contrast to the non-ARDS group (*P* < 0.05) (Figure 1).

### 3.3. Differential Diagnosis Value of Autophagy-Related Protein in Septic Patients with ARDS

**Figure 2:**
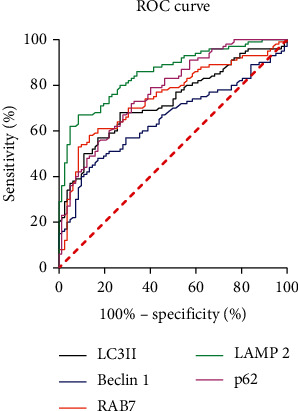
ROC curve of autophagy-associated protein in the diagnosis of sepsis with ARDS.

The results of the ROC curve analysis are shown in Figure 2. The differential diagnosis value of five autophagy-associated proteins for sepsis sufferers with ARDS from high to low is LAMP2, p62, RAB7, LC3II, and Beclin-1, and the specific parameters are shown in Table 2. As shown in Table 2, the AUCs of LAMP2, p62, RAB7, LC3II, and Beclin-1 for the diagnosis of sepsis with ARDS are 0.853, 0.773, 0.746, 0.728, and 0.650, respectively.

### 3.4. The Association between Autophagy-Related Protein and Death in Sepsis Patients with ARDS

**Figure 3:**
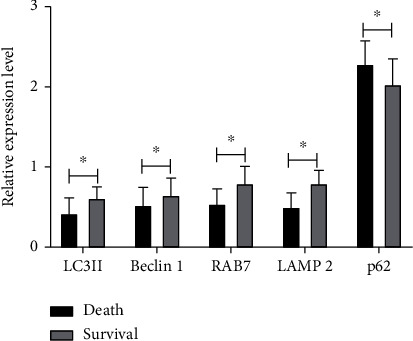
The association between autophagy-related proteins and the death of sepsis patients with ARDS. ^∗^*P* < 0.05.

Among the 82 sepsis sufferers with ARDS included in this research, the sufferers were separated into 2 groups as per whether there were deaths during hospitalization. Among them, there were 38 sufferers in the death group and 44 individuals in the survival group, with a mortality rate of 46.43%. The analysis found that the relative expression levels of LC3II, Beclin-1, RAB7, and LAMP2 in the mortality group were lower in contrast to the survival one (*P* < 0.05), whereas p62 was greater in contrast to the survival one (*P* < 0.05) (Figure 3).

### 3.5. The Prediction Significance of Autophagy-Related Protein in the Death of Sepsis Patients with ARDS

**Figure 4:**
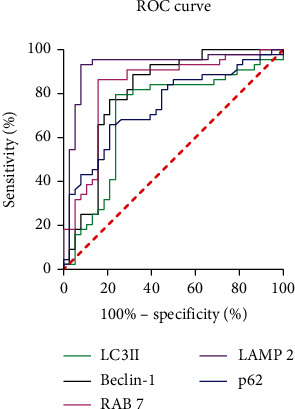
The ROC curve of autophagy-associated protein predicting the death of sepsis sufferers with ARDS.

The results of the ROC curve analysis of autophagy-related proteins predicting the death of sepsis sufferers with ARDS are shown in Figure 4. The predictive value from high to low is LAMP2, RAB7, LC3II, p62, and Beclin-1, and the specific parameters are shown in Table 3. As shown, LAMP2, RAB7, LC3II, p62, and Beclin-1 predicted the AUC of death in sepsis patients with ARDS: 0.922, 0.833, 0.807, 0.745, and 0.708, respectively.

### 3.6. Analysis of Risk Factors for Death in Sepsis Patients with ARDS

Our team further studied the risk factors for mortality in sepsis sufferers with ARDS and incorporated all indicators into the logistics regression model for univariable and multivariable analyses. The outcomes of the univariable analyses are shown in Table 4. Age, CRP, PCT, APACHE II, SOFA, LC3II, Beclin-1, RAB7, LAMP2, and p62 are related to the death of patients with sepsis and ARDS. These indicators are further increased. For factor analysis, the results are shown in Table 5. In the end, only age and SOFA score were discovered to be independent risk factors for mortality in sepsis sufferers with ARDS.

## 4. Discussion

The attributable mortality of ARDS in sepsis sufferers is the most common risk factor for ARDS. Compared with sepsis sufferers without ARDS or non-sepsis-related ARDS, sepsis morbidity and mortality of toxicosis-related ARDS have increased significantly, even as high as 60% [20, 21]. After decades of research and a large number of preclinical and clinical trials, sepsis and ARDS still have no specific and effective drug treatments and basically supported management [22, 23]. Therefore, early diagnosis is essential to ameliorate the prognosis of sepsis sufferers.

In recent years, the autophagic causal links in sepsis sufferers have drawn more and more attention. The autophagic process includes autophagosome forming, autophagosomal-lysosomal fusion, and decomposition products [24]. The autophagic process not only realizes the elimination of damaged protein and cell organs but also realizes the elimination of microbes and causative agents in cytoplasm [25]. In sepsis sufferers, autophagic processes have always been considered an adaptive protection process limiting cellular injury and programmed cell death [26]. ARDS is a serious form of ALI. Pulmonary damage is one of the most commonly seen complicating diseases of sepsis sufferers clinically. It is featured by the releasing of substantial inflammation factors, accompanied by an elevation of programmed cell death and lung oedema. Studies have found that induction of autophagy can protect mice from LPS and mechanical ventilation-triggered ALI and improve artery oxygenation and vessel functions [27]. However, there are currently few clinical studies on autophagy sepsis sufferers with ARDS, and there is a lack of relevant clinical research evidence on whether autophagy-related markers have an effect on the early recognition and prognostic evaluation of sepsis complicated by ARDS.

Herein, our team assessed the diagnostic and prognostic significance of 5 autophagy-related proteins in sepsis complicated by ARDS. The expression of LC3II, Beclin-1, RAB7, and LAMP2 in sepsis patients with ARDS was significantly reduced; p62 was significantly increased, which suggests that autophagy was inhibited in patients with sepsis and ARDS. Further ROC curve analysis results showed that autophagy-related proteins have a higher ability to recognize sepsis with ARDS, and the best one was LAMP2, which had a specificity of 91.46%. Biomarkers can not only be used for early diagnosis of diseases but also predict the clinical outcome of patients. In this study, 38 deaths occurred in 82 patients with sepsis and ARDS, with a mortality rate of 46.34%. We found that the autophagy level in the patients who died was further inhibited, and the surviving patients had LC3II, Beclin-1, RAB7, and LAMP2 levels significantly higher than those in dead patients, and p62 was lower than that in dead patients. ROC analysis results show that the most valuable autophagy-related protein that predicts the death of sepsis sufferers with ARDS is still LAMP2. In the analysis of risk factors for mortality in sepsis sufferers with ARDS, only age and SOFA score were found to be independent risk factors. Previous studies indicated that environmental pollution, vitamin D deficiency, and smoking history may associate with ARDS. However, in our study, due to the limitation of relative information, we could not observe such associations [28]. LAMP2 is a 410 AA, greatly glycosylated protein, which along with LAMP1 accounts for approximately half of the lysosomal membranous protein. LAMP2 participates in lysosome biogenesis and mediates the fusion of lysosomes and autophagosomes [29]. A clinical data study in 2018 found that [29] increased lysosomal gene expression during sepsis was related to unsatisfactory prognostic results. The 5 genes they found that increased in death patients were ATP6AP1, CD63, LAMP1, LAMP2, and SLC1A1. This result is exactly the opposite of ours. The specific reasons have yet to be analyzed. Among their research subjects, they selected patients with SIRS and sepsis suspected of infection in the medical intensive care unit. There was a certain difference with us, and we had not found their sample collection time. In addition, their detection object was a lysosomal gene, which was not consistent with the protein we detected. During the process of gene translation into protein, it will be interfered with by many factors. But these speculations need further research and verification.

In this study, we did observe associations between several biomarkers and the risk of ARDS among sepsis patients. Thus, for the general population with sepsis, these results suggest that carefully and continually monitoring these biomarkers among patients with sepsis is important. Considering the additional benefits of these biomarkers in predicting the risk for long-term morbidity and mortality after sepsis patients comorbid with ARDS, monitoring these biomarkers in clinical practice is necessary.

In conclusion, the autophagy level is significantly inhibited in sepsis patients with ARDS, and autophagy-associated proteins LC3II, Beclin-1, RAB7, LAMP2, and p62 have good value for the diagnosis and prognosis evaluation of sepsis patients with ARDS.

## Figures and Tables

**Table 1:** Analyses of clinic data of two groups of patients.

	ARDS (*n* = 82)	Non-ARDS (*n* = 100)	*χ* ^2^/*t*	*P*
Gender			0.533	0.465
Male	39 (47.56)	53 (53.00)		
Female	43 (52.44)	47 (47.00)		
Age	55.96 ± 8.49	58.01 ± 9.45	1.774	0.078
BMI (kg/m^2^)	23.15 ± 4.04	22.88 ± 3.64	0.475	0.635
Smoking history			10.957	0.001
Yes	48 (58.54)	34 (34.00)		
No	34 (41.46)	66 (66.00)		
Primary disease			0.314	0.997
Severe pneumonia	20 (24.39)	24 (24.00)		
Severe pancreatitis	17 (20.73)	20 (20.00)		
Abdominal infection	16 (19.51)	19 (19.00)		
Biliary infection	12 (14.63)	14 (14.00)		
Surgical trauma	10 (12.19)	12 (12.00)		
Others	7 (8.54)	11 (11.00)		
Chronic complications			2.705	0.439
COPD	26 (31.71)	16 (16.00)		
Cardiac disease	30 (36.58)	35 (35.00)		
Chronic renal failure	12 (14.63)	10 (10.00)		
Liver cirrhosis	24 (29.27)	19 (19.00)		
Creatinine (mmol/l)	125.59 ± 44.59	104.18 ± 30.92	3.812	<0.001
Albumin (g/l)	27.96 ± 4.21	29.14 ± 4.46	1.833	0.068
White blood cell count	18.15 ± 10.48	15.68 ± 8.52	1.751	0.082
CRP	128.17 ± 30.78	97.00 ± 27.56	7.200	<0.001
PCT	15.22 ± 4.15	16.12 ± 4.22	1.445	0.150
APACHE II score	18.81 ± 2.95	13.54 ± 2.24	13.685	<0.001
SOFA score	9.44 ± 3.87	8.46 ± 3.78	1.724	0.086

**Table 2:** The differential diagnosis value of autophagy-related proteins in sepsis sufferers with ARDS.

	AUC	95% CI	*P*	Sensitivity (%)	Specificity (%)	Diagnostic level
LC3II	0.728	0.656-0.801	<0.001	68.00	73.17	<0.631
Beclin-1	0.650	0.570-0.729	0.001	48.00	82.93	<0.815
RAB7	0.746	0.675-0.818	<0.001	58.00	86.59	<0.888
LAMP2	0.853	0.799-0.906	<0.001	67.00	91.46	<0.926
p62	0.773	0.706-0.839	<0.001	72.00	68.29	>1.998

**Table 3:** The predictive value of autophagy-associated protein for the death of sepsis sufferers with ARDS.

	AUC	95% CI	*P*	Sensitivity (%)	Specificity (%)	Diagnostic level
LC3II	0.807	0.706-0.909	<0.001	88.64	68.42	<0.465
Beclin-1	0.708	0.588-0.829	0.001	79.55	76.32	<0.592
RAB7	0.833	0.739-0.928	<0.001	86.36	84.21	<0.639
LAMP2	0.922	0.850-0.993	<0.001	93.18	92.11	<0.646
p62	0.745	0.638-0.852	<0.001	65.91	78.95	>2.088

**Table 4:** Univariate analyses of death in sepsis sufferers with ARDS.

	*B*	S.E.	Wals	df	Sig.	Exp (*B*)	95% CI	
							Lower limit	Upper limit
Sex	-0.610	0.449	1.842	1	0.175	0.543	0.225	1.311
Age	0.114	0.034	11.479	1	0.001	1.121	1.049	1.198
BMI	0.084	0.057	2.177	1	0.140	1.088	0.973	1.217
Smoking history	0.563	0.456	1.523	1	0.217	1.756	0.718	4.293
Primary severe pneumonia	0.194	0.515	0.142	1	0.706	1.214	0.443	3.331
Primary severe pancreatitis	0.334	0.546	0.374	1	0.541	1.397	0.479	4.074
Primary abdominal infection	0.495	0.562	0.776	1	0.378	1.640	0.546	4.932
Primary biliary infection	-0.222	0.633	0.123	1	0.726	0.801	0.232	2.767
Primary surgical trauma	-1.386	0.825	2.824	1	0.093	0.250	0.050	1.259
Other primary diseases	1.157	0.868	1.777	1	0.183	3.182	0.580	17.452
Complicated with COPD	0.671	0.481	1.947	1	0.163	1.957	0.762	5.022
Complicated with cardiac disease	-0.191	0.461	0.172	1	0.678	0.826	0.334	2.040
Complicated with chronic renal failure	0.172	0.625	0.076	1	0.783	1.187	0.349	4.044
Complicated with liver cirrhosis	-1.025	0.521	3.881	1	0.049	0.359	0.129	0.995
Creatinine	0.001	0.005	0.010	1	0.920	1.001	0.991	1.010
Albumin	-0.033	0.053	0.377	1	0.539	0.968	0.872	1.074
White blood cell count	0.018	0.021	0.696	1	0.404	1.018	0.976	1.062
CRP	0.036	0.009	14.894	1	0.000	1.037	1.018	1.056
PCT	0.342	0.083	17.140	1	0.000	1.408	1.197	1.655
APACHE II	0.568	0.131	18.716	1	0.000	1.764	1.364	2.282
SOFA	0.583	0.131	19.646	1	0.000	1.791	1.384	2.317
LC3II	-7.104	1.760	16.302	1	0.000	0.001	0.000	0.026
Beclin-1	-2.832	1.138	6.196	1	0.013	0.059	0.006	0.548
RAB7	-7.891	1.871	17.787	1	0.000	0.000	0.000	0.015
LAMP2	-11.109	2.318	22.962	1	0.000	0.000	0.000	0.001
p62	2.912	0.887	10.783	1	0.001	18.401	3.235	104.659

**Table 5:** Multivariate analyses of death in sepsis sufferers with ARDS.

	*B*	S.E.	Wals	df	Sig.	Exp (*B*)	95% CI	
							Lower limit	Upper limit
Age	0.154	0.068	5.152	1	0.023	1.166	1.021	1.332
CRP	0.001	0.021	0.002	1	0.961	1.001	0.961	1.042
PCT	0.123	0.133	0.854	1	0.356	1.131	0.871	1.468
APACHE II	0.395	0.273	2.098	1	0.148	1.485	0.870	2.534
SOFA	0.695	0.248	7.881	1	0.005	2.004	1.233	3.255
LC3II	0.313	3.156	0.010	1	0.921	1.367	0.003	663.833
Beclin-1	6.623	3.399	3.796	1	0.051	751.852	0.961	588117.797
RAB7	-4.394	3.894	1.273	1	0.259	0.012	0.000	25.502
LAMP2	-4.721	3.328	2.012	1	0.156	0.009	0.000	6.059
p62	-2.896	2.514	1.327	1	0.249	0.055	0.000	7.624

## Data Availability

The data used to support the findings of this study are available from the corresponding author upon request.

## Abbreviation

ARDS: Acute respiratory distress syndrome
LC3: Light chain 3
RAB7: Ras-related protein Rab-7a
LAMP2: Lysosomal-associated membrane protein 2
ALI: Acute lung injury
CLP: Cecal ligation perforation
SIRS: Systemic inflammatory response syndrome
WB: Western blot
CST: Cell Signaling Technology
PVDF: Polyvinylidene fluoride
RT: Room temperature
AA: Amino acid
ARF: Acute respiratory failure
PAGE: Polyacrylamide gel electrophoresis
UVRAG: UV radiation resistance-associated gene protein.
